# Case Report: X-Linked SASH3 Deficiency Presenting as a Common Variable Immunodeficiency

**DOI:** 10.3389/fimmu.2022.881206

**Published:** 2022-04-08

**Authors:** Moisés Labrador-Horrillo, Clara Franco-Jarava, Marina Garcia-Prat, Alba Parra-Martínez, María Antolín, Sandra Salgado-Perandrés, Aina Aguiló-Cucurull, Mónica Martinez-Gallo, Roger Colobran

**Affiliations:** ^1^Allergy Section, Internal Medicine Department, Vall d’Hebron University Hospital (HUVH), Vall d’Hebron Research Institute (VHIR) RETIC ARADyal, Vall d’Hebron Barcelona Hospital, Autonomous University of Barcelona (UAB), Barcelona, Spain; ^2^Immunology Division, Vall d’Hebron University Hospital (HUVH), Vall d’Hebron Barcelona Hospital, Barcelona, Spain; ^3^Translational Immunology Group, Vall d’Hebron Research Institute (VHIR), Vall d’Hebron Barcelona Hospital, Barcelona, Spain; ^4^Department of Cell Biology, Physiology and Immunology, Autonomous University of Barcelona (UAB), Bellaterra, Spain; ^5^Jeffrey Modell Diagnostic and Research Center for Primary Immunodeficiencies, Barcelona, Spain; ^6^Pediatric Infectious Diseases and Immunodeficiencies Unit, Vall d’Hebron University Hospital (HUVH), Vall d’Hebron Barcelona Hospital, Barcelona, Spain; ^7^Infection in Immunocompromised Pediatric Patients Research Group, Vall d’Hebron Research Institute (VHIR), Vall d’Hebron Barcelona Hospital, Barcelona, Spain; ^8^Department of Clinical and Molecular Genetics, Vall d’Hebron University Hospital (HUVH), Vall d’Hebron Barcelona Hospital, Barcelona, Spain

**Keywords:** primary immunodeficiencies, inborn errors of immunity, SASH3 deficiency, common variable immunodeficiency, combined immunodeficiency, genetics, mutation

## Abstract

SASH3 is a lymphoid-specific adaptor protein. In a recent study, SASH3 deficiency was described as a novel X-linked combined immunodeficiency with immune dysregulation, associated with impaired TCR signaling and thymocyte survival in humans. The small number of patients reported to date showed recurrent sinopulmonary, cutaneous and mucosal infections, and autoimmune cytopenia. Here we describe an adult patient previously diagnosed with common variable immunodeficiency (CVID) due to low IgG and IgM levels and recurrent upper tract infections. Two separate, severe viral infections drew our attention and pointed to an underlying T cell defect: severe varicella zoster virus (VZV) infection at the age of 4 years and bilateral pneumonia due type A influenza infection at the age of 38. Genetic testing using an NGS-based custom-targeted gene panel revealed a novel hemizygous loss-of-function variant in the *SASH3* gene (c.505C>T/p.Gln169*). The patient’s immunological phenotype included marked B cell lymphopenia with reduced pre-switch and switch memory B cells, decreased CD4^+^ and CD8^+^ naïve T cells, elevated CD4^+^ and CD8^+^ T_EMRA_ cells, and abnormal T cell activation and proliferation. The patient showed a suboptimal response to *Streptococcus pneumoniae* (polysaccharide) vaccine, and a normal response to *Haemophilus influenzae* type B (conjugate) vaccine and SARS-CoV-2 (RNA) vaccine. In summary, our patient has a combined immunodeficiency, although he presented with a phenotype resembling CVID. Two severe episodes of viral infection alerted us to a possible T-cell defect, and genetic testing led to SASH3 deficiency. Our patient displays a milder phenotype than has been reported previously in these patients, thus expanding the clinical spectrum of this recently identified inborn error of immunity.

## Introduction

Common variable immunodeficiency (CVID) is the most frequently diagnosed primary immunodeficiency in adults. It is characterized by overt hypogammaglobulinemia (low IgG and IgA levels, with or without IgM), and a poor or absent antibody response to infection and immunization. The clinical manifestations of CVID are heterogeneous, and include recurrent respiratory tract infections and other complications related to immune dysregulation, such as gastrointestinal, autoimmune, and lymphoproliferative disorders. Genetically, the heterogeneity is even greater, and during the last decade the increasing use of next-generation sequencing (NGS) technology has accelerated the discovery of novel genes associated with a CVID phenotype, *via* autosomal dominant or recessive inheritance. The immunological consequences of some of these genetic defects clearly go beyond B cell involvement, and this has progressively faded out the boundaries between CVID and combined immunodeficiency (CID). One example is *NFKB1* haploinsufficiency, identified in 2015 and considered the most common monogenic cause of CVID (1, 2). Several reports provide evidence that *NFKB1* haploinsufficiency also underlies a variable type of CID affecting both the B and T lymphocyte compartments, with a broadened spectrum of disease manifestations, including Epstein Barr virus (EBV)-induced lymphoproliferative disease (3–5). A similar situation is seen in *LRBA* deficiency and *CTL4* haploinsufficiency. These two genetic defects have been repeatedly identified in patients initially diagnosed as having CVID and they are usually included in the gene list to be tested in CVID patients (6–8). However, both disorders display a complex variety of clinical and immunologic abnormalities, in most cases involving T cell defects (9).

Because of the specificity and the clinical consequences of these and other genetic defects associated with CVID, once the genetic diagnosis has been established in a patient previously considered to have CVID, the diagnosis should be changed to the respective genetic cause (eg, to *NFKB1* deficiency or *LRBA* deficiency) (PMID:34153571). Still, it is important to keep in mind the various genetic etiologies that can underlie the CVID diagnosis.

Very recently, Delmonte et al. described a novel X-linked CID caused by *SASH3* loss-of-function (LOF) mutations (10). The study included four unrelated patients with recurrent sinopulmonary, cutaneous and mucosal infections, and refractory autoimmune cytopenia. Although all patients were genetically diagnosed in the adulthood, their first clinical manifestations occurred in the childhood. The authors demonstrated that SASH3 deficiency cause a global defect on TCR signaling, which was already evident in T cell progenitors. This leads not only to a reduced numbers of CD3^+^ TCRαβ^+^ cells in the patients, but also to an enrichment of TCR clonotypes with molecular signatures of self-reactivity. Moreover, T cells exhibited functional defects as decreased proliferation and cell cycle progression and increased apoptosis in response to mitogens (10).

Here, we describe a patient initially diagnosed with CVID who presented a novel hemizygous LOF variant in the *SASH3* gene. Our patient displays a milder phenotype than that of the reported patients, thus expanding the clinical spectrum of this novel inborn error of immunity (IEI).

## Case Description

A 43-year-old man, the only child of non-consanguineous parents, attended our allergy clinic with a history of peach allergy (oral allergy syndrome) from childhood. A few years before, the patient had been diagnosed of possible CVID in other center due to low IgG, low IgM, and recurrent upper tract infections. CT sinus scanning showed chronic rhinosinusitis that required 2 to 3 antibiotic courses per year during the following two years, without hospital admission. The chest CT study was normal. Immunoglobulin replacement therapy was not prescribed at that time. During the patient’s clinical follow-up in our hospital, he received a diagnosis of nonspecific lipid transfer protein (nsLTP)-mediated peach allergy. Low IgG levels and absent IgM were confirmed (**Table 1**). A meticulous anamnesis revealed other infectious episodes before the CVID diagnosis, including a severe varicella zoster virus (VZV) infection at the age of 4 that required hospitalization. During adolescence, he experienced mild warts on the hands that did not require treatment and resolved in 3 to 4 years. The patient also mentioned bilateral pneumonia due to type A influenza infection that had required ICU admission (6 days) 5 years previously (at age 38 years). There was no family history of severe infections.

**Table 1:** Immunological parameters of SASH3-deficient patient.

Parameter	Patient values	Reference Values
Leukocytes (x10^9^/L)	7.85	4-11
Lymphocytes (% | x10^9^/L)	**↓ 17.1** | 1.3	20 - 50 | 1.2 - 3.5
CD3+ (% | x10^9^/L)	75.4 | 0.9	55 - 83 | 0.7 - 2.1
CD3+CD4+ (% | x10^9^/L)	37.3 | 0.4	28 - 57 | 0.3 - 1.4
CD3+CD8+ (% | x10^9^/L)	36.7 | 0.4	10 - 39 | 0.4 - 2.1
CD19+ (% | x10^9^/L)	**↓ 3.4 | 0.04 ↓**	6 - 19 | 0.1 - 0.5
CD56+CD3- (% | x10^9^/L)	18.5 | 0.2	7 - 31 | 0.1 - 0.6
CD3+CD4+HLA-DR+ (%)	**↑ 21**	0 - 5
CD3+CD8+HLA-DR+ (%)	**↑ 38**	0 - 5
CD4+ naive (CD3+CD4+CD45RA+CCR7+) (%)	**↓ 12**	29.5 - 50
CD4+ cent. memory (CD3+CD4+CD45RA-CCR7+) (%)	**↑ 29**	14.5 - 27
CD4+ TEMRA (CD3+CD4+CD45RA+CCR7-) (%)	**↑ 20**	4 - 12
CD4+ eff. memory (CD3+CD4+CD45RA-CCR7-) (%)	39	23.5 - 40
CD8+ naive (CD3+CD8+CD45RA+CCR7+) (%)	**↓ 14**	27 - 50
CD8+ cent. memory (CD3+CD8+CD45RA-CCR7+) (%)	1	0.67 - 5.2
CD8+ TEMRA (CD3+CD8+CD45RA+CCR7-) (%)	**↑ 66**	14.5 - 38
CD8+ eff. memory (CD3+CD8+CD45RA-CCR7-) (%)	**↓ 19**	23 - 41.5
Th1 (CD3+CD4+CXCR3+CCR6-) (%)	21	17 - 29
Th17 (CD3+CD4+CCR6+CXCR3-) (%)	**↑ 16**	5 - 12
Th2 (CD3+CD4+ CXCR3-CCR6-) (%)	**↑ 20**	7 - 16
Tfh (CD3+CD4+CXCR5+PD1+)	**↓ 0.5**	16 - 24
Tregs (CD4+CD25^high^CD127-) (%)	3.07	3 - 10
CD19+ transitional (CD24^high^CD38^high^) (%)	8.5	2.4 - 11
CD19+ naive (IgD+CD27-) (%)	**↑ 80**	49 - 72
CD19+ pre-switch memory (IgD+CD27+) (%)	**↓ 1.25**	> 13
CD19+ switch memory (IgD-CD27+) (%)	**↓ 6**	10.1 - 22.2
CD19+ CD21^low^ (%)	2.3	2 - 6
CD19+ plasmablasts^a^ (%)	**↓ 2**	4 - 17
CD69+ expression after PHA	**Low** (45%)	63 - 90
CD69+ expression after anti-CD3/anti-CD28	**Low** (30%)	32 - 80
Lymphocyte proliferation response to PHA	Normal	
Lymphocyte proliferation response to anti-CD3/anti-CD28	Normal	
Lymphocyte proliferation response to PMA + ionomycin	**Very low**	
IgG (mg/dL)	**↓ 425**	700 - 1600
IgA (mg/dL)	159	70 - 400
IgM (mg/dL)	**↓ <20**	40 - 230
IgE (KU/L)	**↑ 266**	0 - 117
IgE specific to Cow milk, f2 (KU/L)	**↑ 0.72**	0 - 0.35
IgE specific to Peach, f95	**↑ 5.78**	0 - 0.35
IgE specific to rPru p3, f420	**↑ 5.34**	0 - 0.35
IgE specific to *D. pteronyssinus*	**↑ 4.2**	0 - 0.35
IgE specific to shrimp, f24	**↑ 2.93**	0 - 0.35
C3 (mg/dL)	145	85 - 180
C4 (mg/dL)	23.3	10 - 40
IgG anti-*H. influenzae* type B (fold-increase)	133.76	>4 fold-increase
IgG anti-*S. pneu*moniae (fold-increase)	**↓ 2.8**	>4 fold-increase
IgG anti-SARS-COV2 (after 1^st^ dose vaccine)	40.9	n.a.
IgG anti-SARS-COV2 (after 2^nd^ dose vaccine)	>800	n.a.
SARS-CoV-2 Quantiferon (after 1^st^ dose vaccine)	Negative	n.a.
SARS-CoV-2 Quantiferon (after 2^nd^ dose vaccine)	Positive	n.a.

a Gated from CD19+ switch memory cells (IgD-CD27+).

Arrows indicate values that are above or below the reference values.

Values that deviate from reference values are indicated in bold.

n.a. means not available.

## Diagnostic Assessment

### Genetic Diagnosis by Next-Generation Sequencing

**Figure 1:**
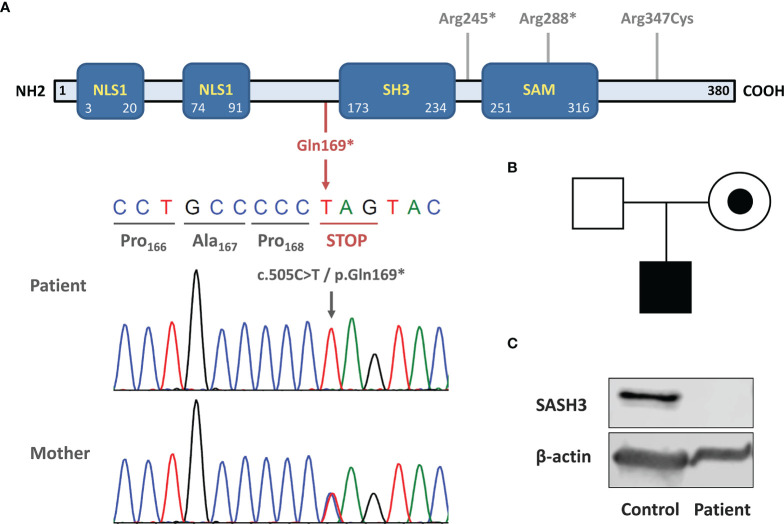
Novel loss-of-function variant causing SASH3 deficiency. **(A)** Schematic structure of SASH3 protein. The location of the c.505C>T/p.Gln169* variant is indicated below in red and Sanger sequencing confirmation is shown. The three other variants reported by Delmonte et al. are indicated in gray in the upper part. **(B)** Pedigree and familial segregation of the *SASH3* variant. The patient was hemizygous and his mother, a heterozygous carrier. **(C)** Western blot showing absence of SASH3 protein in patient PBMCs. The immunogen recognized by the antibody used (SASH3 polyclonal antibody #PA5-70305, Termofisher Scientific) is located at the N-terminal region of human SASH3 protein, before the amino acid 169 (where the STOP codon is located).

Although the patient was initially diagnosed with CVID, the history of severe viral infections was an alert sign pointing to possible CID. He was tested using our NGS-based custom-targeted gene panel including 427 IEI-associated genes. We found a novel hemizygous variant in *SASH3*, consisting of a single nucleotide change (c.505C>T) leading to a premature STOP codon (p.Gln169*). This is a private variant (not present in the main population databases) and is predicted to cause LOF by truncating the SASH3 protein upstream of the SAM and SH3 domains. To date only three *SASH3* variants have been reported, two nonsense and one missense, with p.Gln169* being the most N-terminal one (**Figure 1A**). The variant was confirmed by Sanger and family study, which showed that the mother is a heterozygous carrier (**Figure 1B**). Western blot of the patient’s PBMCs using a polyclonal antibody targeting the N-terminus of SASH3 showed complete absence of protein, thus confirming the LOF nature of the c.505C>T/p.Gln169* variant (**Figure 1C**).

### Immune System Evaluation

**Figure 2:**
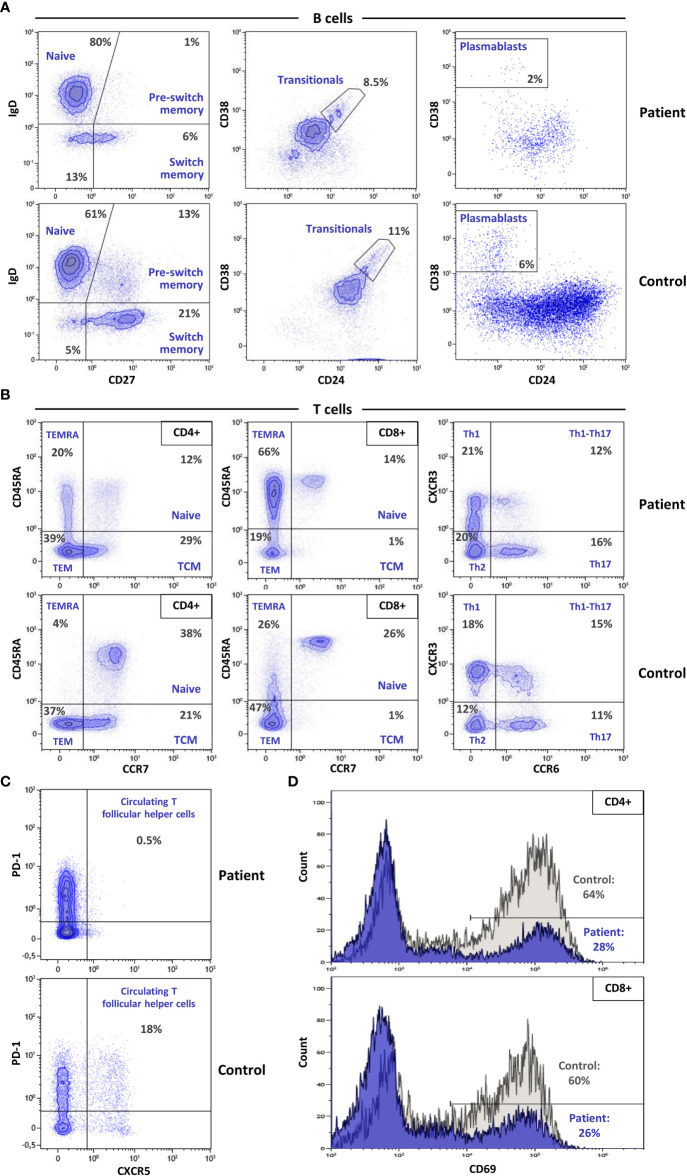
Multicolor flow cytometry evaluation of advanced lymphocyte subpopulations and T cell activation. **(A)** Dot plots illustrating the main differences in B cell subpopulations between the patient and a healthy control show a dramatic reduction in pre-switch, isotype switching, and plasmablast populations in the patient. **(B)** Advanced T lymphocyte analysis shows decreased naïve CD4 and CD8 populations, with a striking increase of lymphocytes with T_EMRA_ phenotype. **(C)** Absence of cTfh cells in the patient vs. the control. **(D)** CD69 expression after activation with anti-CD3/CD28 in CD4 (upper) and CD8 (lower) lymphocytes, depicts decreased activation capacity in the SASH3-deficient patient.

After the genetic finding of SASH3 deficiency, recently reported as a new type of CID (10), we aimed to assess the patient’s immunological phenotype. White blood cell count was normal, but he had marked B cell lymphopenia both in percentage and absolute numbers (**Table 1**). Most B cells were naïve, with reductions in pre-switch and switch memory B cells (**Figure 2A**). Plasmablasts were decreased, whereas CD19^+^CD21^low^ B cells (usually expanded in autoimmune conditions) were in the normal range.

T cell numbers were normal in both the CD4^+^ and CD8^+^ compartments, but analysis of T lymphocyte subset distributions demonstrated a marked decrease in CD4^+^ and CD8^+^ naïve T cells (**Figure 2B**; **Table 1**). Conversely, CD4^+^ and CD8^+^ T_EMRA_ cells were elevated, more markedly in the case of CD8^+^ T cells, and were accompanied by an increase in HLA-DR expression. There was a complete absence of circulating T follicular helper (cTfh) cells (**Figure 2C**). Distributions of Th1, Th2, Th17, Tregs, and NK cells were in the normal range.

CD4^+^ and CD8^+^ T cells both showed low CD69 expression on stimulation with anti-CD3 and anti-CD28, indicating defective activation (**Figure 2D**). *In vitro* T-cell proliferation was also tested. Results were normal when using PHA and anti-CD3/anti-CD28 stimulation, but proliferation was very low in response to PMA + ionomycin (**Table 1**), indicating that distal intracellular signaling may have been affected.

Finally, vaccine responsiveness was evaluated. The patient showed a suboptimal response to *Streptococcus pneumoniae* (polysaccharide) vaccine and a normal response to *Haemophilus influenzae* type B (conjugate) vaccine. There was a good humoral and cellular response to SARS-CoV-2 vaccine (BNT162b2, Pfizer-BioNTech), especially after the second dose (**Table 1**).

## Discussion

SASH3 (also known as SH3-containing lymphocyte protein, SLY1) is a lymphoid-specific adaptor protein. SASH3 deficiency, recently described by Delmonte et al., is a novel combined immunodeficiency with immune dysregulation, associated with impaired TCR signaling and thymocyte survival in humans (10). These authors identified four unrelated males carrying three novel deleterious hemizygous LOF variants in the *SASH3* gene. The present study is the second to date describing a patient with SASH3 deficiency. Our patient carried the c.505C>T/p.Gln169* variant, which truncates SASH3 protein upstream of the SAM and SH3 domains and leads to a complete absence of the protein.

Comparison of the four patients reported by Delmonte et al. and our patient shows some similarities and differences (**Table 2**). At first glimpse, our patient experienced a less severe clinical course. He was diagnosed with possible CVID at the age of 41 years due to low IgG and IgM levels and recurrent upper tract infections (with a normal chest CT scan). But up to now, he has not shown autoimmune cytopenia or other forms of autoimmunity, and he has no relevant skin manifestations (severe warts or infections) or signs of lymphoproliferation. However, the history of two pertinent viral infections, severe VZV infection in childhood and bilateral pneumonia due type A influenza infection at the age of 38 years, indicated a possible underlying T cell defect. Indeed, as in the previously reported cases, our patient showed a marked decrease in CD4 and CD8 naïve T cells with defective T cell activation and proliferation. Strikingly, whereas the four patients described by Delmonte et al. had severe NK cell lymphopenia, our patient had normal NK counts (**Table 2**).

**Table 2:** Clinical and immunological features of all reported SASH3 deficient patients.

	P1 (Delmonte et al.)	P2 (Delmonte et al.)	P3 (Delmonte et al.)	P4 (Delmonte et al.)	P5 (This study)
Sex	Male	Male	Male	Male	Male
Age at diagnosis, years	19	50	27	56	41
Age at onset, years	3	5	2	n.a.	4
**Clinical manifestations**					
Viral infections	Yes	–	Yes	Yes	Yes
Bacterial infections	Yes	Yes	Yes	Yes	Yes
Fungal infections	–	–	Yes	–	–
Autoimmunity	Yes	Yes	Yes	Yes	–
Skin manifestations	Yes	–	Yes	Yes	–
Lymphoproliferation	–	Yes	Yes	–	–
**Laboratory information**					
Anemia	–	Yes	Yes	–	–
Neutropenia	Yes	Yes	Yes	–	–
Lymphopenia	–	Yes	–	Yes	–
Thrombocytopenia	Yes	–	Yes	–	–
IgG	normal	IVIG	IVIG	↓	↓
IgA	normal	↓	↓	↓	normal
IgM	↓	↑	↓	↓	↓
Response to vaccines	Yes	n.a.	n.a.	Yes	Yes^a^
T cells	normal	↓	normal	↓	normal
CD4	↓	normal	↓	↓	normal
CD4 naïve	↓	↓	↓	↓	↓
CD8	↑	↓	↑	normal	normal
CD8 naive	normal	↓	normal	↓	↓
CD8 TEMRA	↑	normal	↑	normal	↑
Th1	↓	normal	↓	↓	normal
Th17	↓	↓	↓	↓	normal
Th2	normal	normal	normal	normal	normal
Treg	↓	↓	↓	normal	normal
B cells	↓↓	↓↓	↓↓	↓↓	↓↓
NK cells	↓↓	↓↓	↓↓	↓↓	normal
*In vitro* TCR activation	↓	↓	↓	↓	↓
*In vitro* lymphoproliferation^b^	↓	↓	↓	↓	↓
SASH3 mutation	p.R347C	p.R288*	p.R288*	p.R245*	p.Q169*

n.a., not available; the symbol “-” means “no”; IVIG, intravenous immunoglobulin.

a Suboptimal response to the S. pneumoniae (polysaccharide) vaccine.

b With anti-CD3 and PMA + ionomycin.

Arrows indicate values that are above or below the reference values.

Regarding B cell status, our patient showed marked B cell lymphopenia with very low pre-switch and low switch memory B cells and plasmablasts, in accordance with findings in the other reported patients. The response to various vaccines was normal in all patients tested, although our patient had a suboptimal response to a polysaccharide vaccine (*S. pneumoniae*). Remarkably, our patient showed a complete absence of cTfh cells, a feature that had not been evaluated in the previous patients. cTfh cells are peripheral counterparts of conventional Tfh cells that are predominantly located in secondary lymphoid tissues and play a crucial role in T cell-dependent humoral immunity and the proper response to most vaccines. Why the response to vaccines was essentially normal in the absence of cTfh is challenging to explain, but the exact correlation between cTfh and successful antibody response remains controversial (11).

Our patient has an oral allergy syndrome with elevated total and allergen-specific IgE that was not described in the previously reported patients (10). It is difficult to establish whether this allergy is related with the SASH3 deficiency or is an independent phenomenon. Nonetheless, oral allergy syndrome is not rare and is likely not a distinctive feature of SASH3 deficiency.

The five SASH3 deficient patients reported to date recapitulate many of the immunological defects described in the two different *Sly1* deficient mice models generated (*Sly1*^Δ/Δ^ and *Sly1*^-/-^) (12, 13). In both models it has been shown that: (1) absence of SLY1 (SASH3) protein affects CD4 T cell development, T-cell proliferation and cytokine production; (2) SLY1 is an anti-apoptotic factor required for thymocyte development and (3) antibody responses to T-dependent and T-independent antigens were impaired. The functional T cell defects identified in *SASH3* deficient patients confirm the crucial role of this lymphocyte-specific factor for T cell development and function in humans.

In summary, our patient has a combined immunodeficiency due to SASH3 deficiency, although he presented with a phenotype initially resembling CVID. Two separate, severe viral episodes alerted us to an underlying T-cell defect. The most relevant difference compared with the patients described so far is the absence of evident immune dysregulation. Undoubtedly, future cases of SASH3 deficiency will help to better define the prevalence of the various clinical and cellular characteristics of this IEI. In the meantime, SASH3 deficiency should be considered as one of the monogenic defects that can occur in male patients presenting with a CVID-like phenotype.

## Data Availability Statement

The original contributions presented in the study are included in the article/supplementary material. Further inquiries can be directed to the corresponding authors.

## Ethics Statement

The studies involving human participants were reviewed and approved by Ethics Review Board of Hospital Universitari Vall d’Hebron. The patients/participants provided their written informed consent to participate in this study. Written informed consent was obtained from the individual(s) for the publication of any potentially identifiable images or data included in this article.

## Author Contributions

ML-H provided patient care and collected clinical data. MG-P and AP-M performed laboratory analysis and western blot under the supervision of CF-J. SS-P performed flow cytometry analysis under the supervision of MM-G. AA-C performed the molecular experiments under the supervision of RC. MA and RC analyzed the NGS data and provided the genetic diagnosis. ML-H, CF-J, MM-G, and RC analyzed and interpreted the data and wrote the manuscript. MM-G and RC designed and supervised the project, provided resources and edited the manuscript. All co-authors reviewed, commented and approved the final version of the manuscript.

## Funding

This study was funded by Instituto de Salud Carlos III, grants PI17/00660 and PI20/00761, cofinanced by the European Regional Development Fund (ERDF).

## Conflict of Interest

The authors declare that the research was conducted in the absence of any commercial or financial relationships that could be construed as a potential conflict of interest.

## Publisher’s Note

All claims expressed in this article are solely those of the authors and do not necessarily represent those of their affiliated organizations, or those of the publisher, the editors and the reviewers. Any product that may be evaluated in this article, or claim that may be made by its manufacturer, is not guaranteed or endorsed by the publisher.
